# Single-nucleotide resolution detection of Topo IV cleavage activity in the *Escherichia coli* genome with Topo-Seq

**DOI:** 10.3389/fmicb.2023.1160736

**Published:** 2023-04-06

**Authors:** Dmitry Sutormin, Alina Galivondzhyan, Azamat Gafurov, Konstantin Severinov

**Affiliations:** ^1^Skolkovo Institute of Science and Technology, Moscow, Russia; ^2^Waksman Institute for Microbiology, Rutgers, The State University of New Jersey, Piscataway, NJ, United States

**Keywords:** *E. coli*, topoisomerase IV, genome topology, Topo-Seq, supercoiling, decatenation

## Abstract

Topoisomerase IV (Topo IV) is the main decatenation enzyme in *Escherichia coli*; it removes catenation links that are formed during DNA replication. Topo IV binding and cleavage sites were previously identified in the *E. coli* genome with ChIP-Seq and NorfIP. Here, we used a more sensitive, single-nucleotide resolution Topo-Seq procedure to identify Topo IV cleavage sites (TCSs) genome-wide. We detected thousands of TCSs scattered in the bacterial genome. The determined cleavage motif of Topo IV contained previously known cleavage determinants (−4G/+8C, −2A/+6 T, −1 T/+5A) and additional, not observed previously, positions −7C/+11G and −6C/+10G. TCSs were depleted in the Ter macrodomain except for two exceptionally strong non-canonical cleavage sites located in 33 and 38 bp from the *XerC-box* of the *dif*-site. Topo IV cleavage activity was increased in Left and Right macrodomains flanking the Ter macrodomain and was especially high in the 50–60 kb region containing the *oriC* origin of replication. Topo IV enrichment was also increased downstream of highly active transcription units, indicating that the enzyme is involved in relaxation of transcription-induced positive supercoiling.

## Introduction

Replication of long DNA molecules, especially, circular chromosomes, leads to accumulation of positive DNA supercoiling (Postow et al., 2001), which, through rotation of the DNA polymerase complex, is thought to lead to formation of precatenanes (overtwisted sister chromosomes) (Peter et al., 1998; Cebrián et al., 2015). If left unresolved, precatenanes interfere with bulk DNA segregation during replication and lead to sister chromosomes catenation after the replication is completed (Adams et al., 1992; Lucas et al., 2001). Specialized enzymes, topoisomerases, resolve such topological challenges and support proper DNA replication and segregation processes (Adams et al., 1992; Khodursky et al., 2000; Schvartzman et al., 2019).

In *Escherichia coli*, topoisomerase III, topoisomerase IV, and DNA gyrase are thought to contribute to the resolution of catenanes and segregation of replicating molecules (Adams et al., 1992; Hiasa et al., 1994; Khodursky et al., 2000; Seol et al., 2013; Lee et al., 2019). While the functions of these enzymes may be partially redundant, topoisomerase IV (Topo IV) is considered to be the main decatenase (Zechiedrich et al., 1997). Topo IV is encoded by the *parC* and *parE* genes and functions as a heterotetrameric complex ParC_2_ParE_2_ (Kato et al., 1990; Peng and Marians, 1993). Topo IV is a classical type-IIA topoisomerase: it introduces a transient double-stranded break in one segment of DNA (called G-segment, for “Gate”) and transfers another DNA segment (called T-segment, for “Transport”) through the break. To maintain the break during the transfer, the enzyme produces 4-nt 5′-overhangs with 5′-ends covalently linked to catalytic ParC Tyr residues located in the DNA-gate of the enzyme (Peng and Marians, 1993). *In vitro* and *in vivo*, Topo IV efficiently decatenates circular DNA molecules and resolves precatenanes (Adams et al., 1992; Zechiedrich and Cozzarelli, 1995; Seol et al., 2013). Topo IV specificity toward DNA crossovers is linked to the structure of the ParC C-terminal domain (CTD) (Hirsch and Klostermeier, 2021). Topo IV relaxes positive supercoiling more efficiently than negative supercoiling and thus may partially substitute for DNA gyrase, another type-IIA topoisomerase that also relieves positive supercoiling and, uniquely, introduces negative supercoils in an ATP-dependent process (Crisona et al., 2000; Khodursky et al., 2000; Ashley et al., 2017).

In *E. coli*, depletion of Topo IV results in a *par* phenotype (Kato et al., 1990): DNA replication rate is mildly inhibited (Khodursky et al., 2000), segregation of replicated DNA is impaired (Wang et al., 2008), genome structure is reorganized (Conin et al., 2022), and the rate of formation of “ghost” cells lacking DNA is increased (Grainge et al., 2007; Wang et al., 2008). Conversely, increased intracellular amounts of Topo IV accelerate segregation of replicated DNA (Wang et al., 2008).

*Escherichia coli* Topo IV has several known protein partners which modulate its activity *via* direct protein–protein interactions with CTDs. The condensin complex MukBEF interacts through its hinge domain with Topo IV (Hayama and Marians, 2010; Li et al., 2010; Vos et al., 2013). *In vitro*, this interaction was reported to stimulate the relaxation and decatenation activities of Topo IV (Hayama and Marians, 2010; Li et al., 2010), though this finding was later disputed (Kumar et al., 2022). *In vivo*, the interaction between MukBEF and Topo IV is necessary for timely segregation of newly replicated regions containing the origin of replication (Nicolas et al., 2014; Zawadzki et al., 2015) and chromosomal DNA (Kumar et al., 2017). At the origin, Topo IV and MukBEF were shown to form clusters consisting of 15–16 Topo IV and MukBEF complexes (Nicolas et al., 2014; Zawadzki et al., 2015; Nolivos et al., 2016). These clusters may act as a scaffold to initiate chromosomal DNA condensation (Vos et al., 2013). *In vitro*, decatenation and relaxation of negative supercoiling by Topo IV were reported to be stimulated by low concentrations of SeqA. The effect appears to be specific, since topoisomerase I or DNA gyrase activities were unaffected. However, high concentrations of SeqA caused non-specific inhibition of activities of all topoisomerases (Kang et al., 2003). SeqA binds hemimethylated DNA and therefore effectively follows the replisome during replication. It is required for temporary cohesion of newly replicated regions, which may be achieved by inhibition of Topo IV activity on SeqA-bound hemimethylated chromosomal regions behind the replisome (Joshi et al., 2013; Helgesen et al., 2021).

The Ter macrodomain (MD) of the *E. coli* chromosome is organized by the MatP protein, which binds to *matS* sites scattered through Ter (Mercier et al., 2008). MatP forms dimers and dimers of dimers, binds MukBEF, and promotes its unloading from DNA (Nolivos et al., 2016). The interaction of MatP and Topo IV with MukBEF was shown to be competitive (Fisher et al., 2021) and it was proposed that MatP-mediated displacement of MukBEF-Topo IV complexes from Ter promotes the turnover of complexes and their loading at the origin (Nolivos et al., 2016). In the middle of Ter, a *dif*-site is located. It is recognized by heterotetrameric resolvase complex XerC/XerD that resolves chromosome dimers to monomers (Blakely et al., 1993). A Topo IV cleavage hot-spot was found in the vicinity of the *dif*-site (Hojgaard et al., 1999; El Sayyed et al., 2016). XerC is required for Topo IV binding and cleavage at this site implying a direct interaction between Topo IV and XerC, which, however, has not yet been demonstrated (El Sayyed et al., 2016). Another essential component of resolvase machinery, an FtsK DNA translocase, also binds Topo IV and stimulates its decatenation and relaxation activities (Espeli et al., 2003; Bigot and Marians, 2010). Considering the FtsK interaction with XerD and activation of recombination by XerC/XerD (Grainge et al., 2011; Keller et al., 2016), a complex multiprotein machine must be operating at the *dif-*site to secure the resolution of chromosome dimers and catenanes.

The Topo IV cleavage specificity was extensively studied *in vitro*. Several symmetrical nucleotide determinants at positions −4G/+8C, −2A/+6 T, −1 T/+5A relative to the left cleavage site located at position +1 were identified as important for cleavage (Leo et al., 2005; Richter et al., 2007; Arnoldi et al., 2013). On a whole-genome level and *in vivo*, Topo IV binding and cleavage were studied by, respectively, ChIP-Seq and NorfIP. The latter method utilizes stabilization of the intermediate covalent cleavage complex between Topo IV and DNA by a topoisomerase poison norfloxacin. With NorfIP, the strong cleavage at *dif* was validated and hundreds of cleavage sites scattered throughout the *E. coli* genome were revealed (El Sayyed et al., 2016). Since NorfIP has a limited resolution, we here decided to map Topo IV cleavage sites with Topo-Seq, a method previously developed in our laboratory. Topo-Seq also employs stabilization properties of topoisomerase poisons to trap covalent complexes of type-II topoisomerases with DNA, but relies on a specific protocol for preparation of sequencing libraries from single-stranded DNA, which allows to map cleavage sites strand specifically and with a single-nucleotide resolution (Sutormin et al., 2019). Using Topo-Seq, we identified several thousand Topo IV cleavage sites in the *E. coli* genome and derived a Topo IV cleavage motif. The motif contains several determinants that were not reported previously and lacks the signs of DNA wrapping observed in the DNA gyrase cleavage motif (Sutormin et al., 2019). On a whole-genome level, Topo IV cleavage sites were significantly depleted in the Ter MD and highly enriched in a 50–60 kb region containing the origin of replication *oriC*. We found two strong non-canonical Topo IV cleavage signals, 33 and 38 bp from the *XerC-box* of the *dif*-site. Finally, we found that Topo IV enrichment was increased downstream of active transcription units, where positive supercoiling is accumulated. These results significantly expand the knowledge about Topo IV activity in the *E. coli* genome. With minimal modification, the Topo-Seq approach can be extended for studies of Topo IV in other bacteria.

## Materials and methods

### Strains and plasmids

*Escherichia coli* DY330 *parC-SPA* strain (W3110 *lacU169 gal490 cI857 Δ*(*cro-bioA*) *parC*-*SPA*) with *parC* gene fused with the sequence encoding the SPA tag (purchased from Horizon Discovery Biosciences) was used in Topo IV Topo-Seq experiments. *E. coli* DY330 *parC-SPA gyrA-S83L* was constructed from the original strain by recombineering. pKD46 plasmid was used for recombineering.

### Introduction of the gyrA-S83L mutation

The *gyrA-S83L* mutation conferring resistance to fluoroquinolone antibiotics (Bagel et al., 1999), was introduced to the *E. coli* DY330 *parC-SPA* strain by recombineering using the template oligonucleotide complementary to the lagging DNA strand (Sawitzke et al., 2011). A culture of *E. coli* DY330 *parC-SPA* harboring the pKD46 plasmid was prepared for recombineering as described in Datsenko and Wanner (2000) and 50 μl aliquot of cells (~6*10^8^ cells) was electroporated with 0.2 μl of 50 μM chemically synthesized 70-mer oligonucleotide (~6*10^12^ molecules) (GCGCCATGCGGACGATCGTGTCATAGACCGCCAGATCACCATGGGGATGG-TATTTACCGATTACGTCACC). Fluoroquinolone-resistant mutants were selected on LB plates containing 500 nM ciprofloxacin. To verify the mutation acquisition, a fragment of *gyrA* gene was PCR amplified using gyrA_F (ATGAGCGACCTTGCGAGAGAAATTACACCG) and gyrA_R (CCGGAATTTTTTCCG-TGCCGTC) primers and subjected to Sanger sequencing.

### MIC estimation

Minimal inhibitory concentrations (MIC) for ciprofloxacin and tetracycline were measured for *E. coli* DY330 *parC-SPA* and *E. coli* DY330 *parC-SPA gyrA-S83L* liquid cultures by a serial dilution method in 200-μL 96-well plates in LB. The plates were incubated at 37°C for 24 h and MIC was defined as a minimal concentration suppressing the growth of a liquid culture.

### Topo IV Topo-Seq

Purification and sequencing of DNA fragments covalently attached to the poisoned Topo IV (Topo-Seq) were performed with *E. coli* DY330 *parC-SPA* and *E. coli* DY330 *parC-SPA gyrA-S83L* as described previously for *E. coli* DNA gyrase (Sutormin et al., 2019). Briefly, ciprofloxacin (final concentration 10 μM) was added to the 50 ml of exponentially growing cell culture in LB medium when OD_600_ ~ 0.6, and the culture was incubated for 15 min with rotation at 37°C. Cells were harvested by centrifugation and washed twice with TES buffer (10 mM Tris–HCl pH 7.5, 1 mM EDTA, 250 mM NaCl). Then, cells were resuspended in 1 ml of TES and disrupted by sonication (DNA fragmentation range 200–800 bp). Lysate was cleared by centrifugation and incubated with 100 μl of ANTI-FLAG M2 affinity gel (Sigma-Aldrich). After immunoprecipitation, the resin was treated with Proteinase K (Sigma-Aldrich) in 200 μl of TES and DNA fragments were purified with AMPure XP magnetic beads from the supernatant (Beckman Coulter). Non-treated control was processed identically, except that ciprofloxacin was not added. For mock control, DNA was extracted from 100 μl of cleared lysate using the GeneJET DNA purification kit (Thermo Scientific).

NGS libraries were prepared using Accel NGS 1S kit (Swift Bioscience). DNA sequencing was performed with Illumina HiSeq 4000 in a 150 + 150 bp paired-end mode. Library preparation and sequencing were performed at Skoltech Genomics Core Facility. Topo-Seq was performed in triplicate.

Raw reads were filtered with Trimmomatic (Bolger et al., 2014) and then aligned to the *E. coli* W3110 MuSGS genome (the genome may be obtained from GEO: GSE95567) using BWA-MEM (Li and Durbin, 2010). SAM, BAM, and bed files were prepared with Samtools (Li et al., 2009). The number of DNA fragments’ 3′-ends (N3E) was calculated per position, based on the read alignments stored in SAM files. Normalization of N3E tracks and identification of Topoisomerase Cleavage Sites (TCSs) were performed as described previously (Sutormin et al., 2019). The resultant tracks were further analyzed using custom Python scripts.^1^ *E. coli* reference genome annotation with transcription units was obtained from the EcoCyc database (Karp et al., 2018).

### Analysis of external NGS datasets

Topo IV ChIP-Seq and NorfIP data were taken from publicly available GEO dataset GSE75641 (El Sayyed et al., 2016). Raw reads were aligned to the *E. coli* W3110 MuSGS reference genome, and alignment was converted to coverage depth tracks using Samtools (Li et al., 2009). Fold enrichment tracks were obtained by position-wise division of IP samples by corresponding mock samples. MatP and MukB ChIP-Seq datasets were taken from publicly available GEO dataset GSE67221 (Nolivos et al., 2016) and processed as described before. 3C data for *wt E. coli* were taken from GSE107301 (Lioy et al., 2018). Contact maps were generated using the pipelines^2^,^3^ (Lioy et al., 2018). *E. coli* W3110 MuSGS reference genome was used for reads alignment. Contact data were filtered with thresholds: 3 restriction fragments for uncuts and 2 restriction fragments for loops. Resultant contact maps were binned at 10 kb resolution. RNA-Seq data were taken from GEO dataset GSE181687 (Sutormin et al., 2022). Full list of NGS datasets used in this study can be found in Supplementary Table 2.

### Plasmid topology analysis by electrophoresis with chloroquine

Plasmid DNA was extracted with GeneJet Plasmid Miniprep Kit (Thermo Fisher) from stationary phase (overnight growth, 5 ml) and exponential phase (OD_600_ = 0.6, 50 ml) cultures of *E. coli* DY330 *parC-SPA* and *E. coli* DY330 *parC-SPA gyrA-S83L* strains cultivated in LB at 37°C. 1.2 μg of plasmid was separated by electrophoresis (120 V) in 1% agarose gel in ice-cold TAE buffer supplemented with 2.5 μg/ml chloroquine and visualized by ethidium bromide staining.

### Microscopy

*Escherichia coli* DY330 *parC-SPA* or *E. coli* DY330 *parC-SPA gyrA-S83L* cells were grown and treated with Cfx as described in the Topo-Seq section. 50-μL aliquots of the cells were stained for 5 min in 20 ng/ml 4′,6-diamidino-2-phenylindole (DAPI). Cells were spotted on agarose pads (1.2% agarose in PBS) and imaged at 100× magnification using a Nikon Eclipse Ti microscope controlled by NIS-Elements BR 4.51.01 and equipped with the Nikon Plan Apo VC 100 × 1.40 oil objective and Nikon DS-Qi2 digital monochrome camera. Images were processed using ImageJ2 v2.35 software (Schneider et al., 2012).

## Results

### Identification of Topo IV cleavage sites

**Figure 1:**
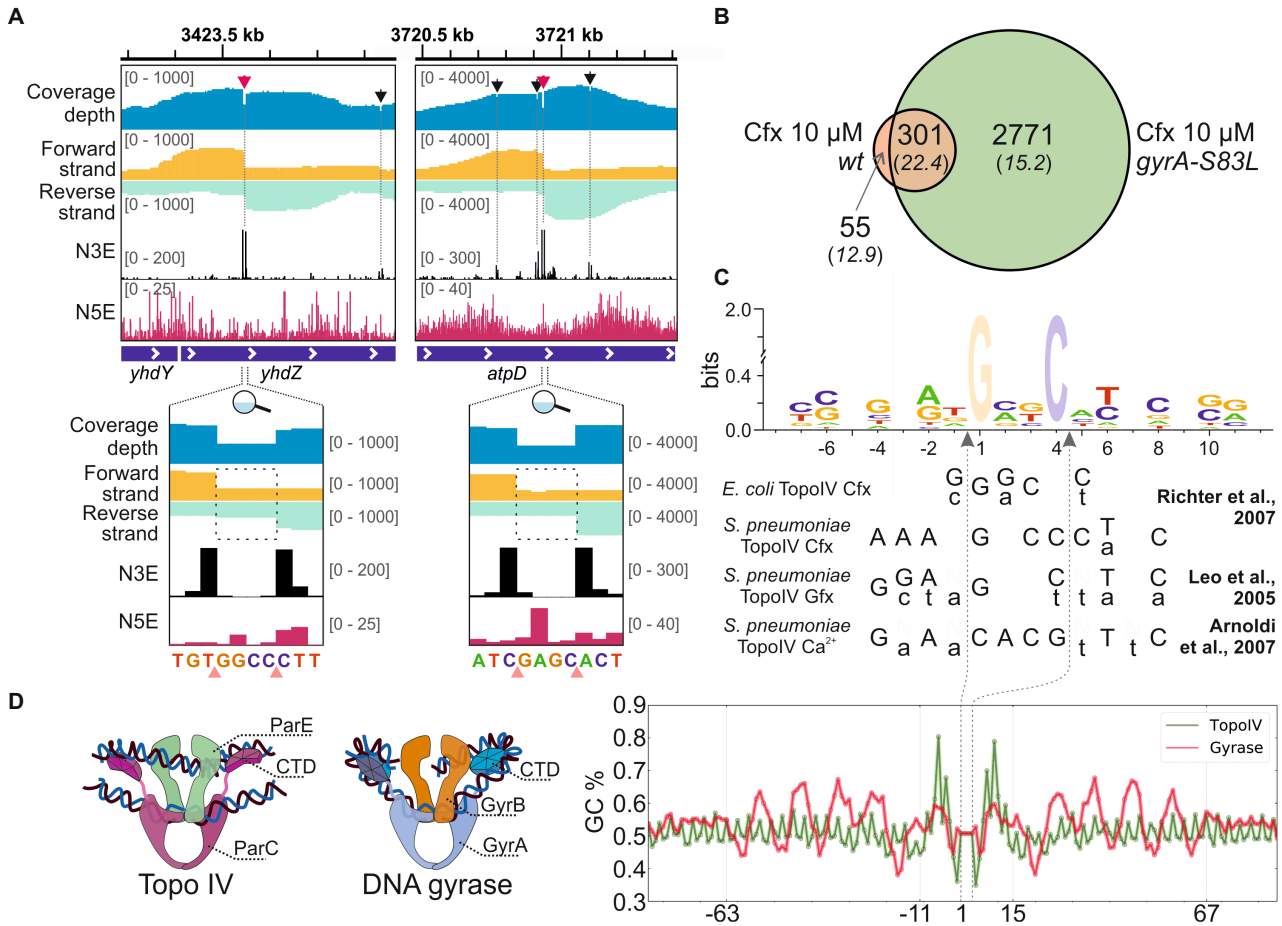
Detection of Topo IV TCSs with Topo-Seq. **(A)** Representative TCSs and cleavage patterns. The total sequencing coverage depth is shown in blue at the top. Coverage depths for forward and reverse strands are shown in gold and turquoise, respectively. Profiles of the number of 5′- (N5E) and 3′-ends (N3E) are shown in black and red, correspondingly. Tracks depths are shown in brackets. TCSs are shown with arrows and dashed lines. Primary and satellite TCSs (see text) are shown with red and black arrows, correspondingly. Zoom-in views of primary cleavage sites are shown below. Cleavage sites are shown with pink triangles. Dashed rectangles mark the gaps between coverage depths on forward and reverse strands. The data were visualized in IGV (Thorvaldsdóttir et al., 2013). **(B)** A Venn diagram representing an overlap between TCSs sets identified for *wt* and *gyrA-S83L* strains. **(C)** Logo representation of Topo IV cleavage motif determined with Topo-Seq. Cfx-biased positions at positions +1 and + 4 are translucent. Below the logo, cleavage consensus sequences identified previously for *E. coli* and *S. pneumonia* Topo IV *in vitro* are shown (Leo et al., 2005; Richter et al., 2007; Arnoldi et al., 2013). Preferred bases are shown in capital letters and non-preferred bases are shown in lowercase letters. Gfx—gemifloxacin. Vertical dashed arrows indicate positions of cleavage sites. **(D)** The Topo IV motif (green) and the DNA gyrase ‘combined’ motif (red) are shown as plots of GC content (right). Cartoon representations of Topo IV and DNA gyrase complexes with G- and T-segment DNAs are shown on the left.

To map the sites of *E. coli* Topo IV activity genome-wide with single-nucleotide resolution, Topo-Seq, a protocol previously developed to monitor genome-wide distribution of *E. coli* DNA gyrase activity (Sutormin et al., 2019), was applied to exponentially growing cultures of *E. coli* DY330 *parC-SPA* strain. This strain encodes an affinity-tagged ParC subunit of Topo IV and behaves as wild-type, i.e., shows no *par* phenotype. We reasoned that the affinity tag should allow us to purify covalent ParC-DNA adducts formed in the presence of Topo IV poison ciprofloxacin (Cfx). While Cfx also targets the DNA gyrase, the adducts of this enzyme shall not be recovered during affinity purification. Three parallel DY330 *parC-SPA* cultures were treated with 10 μM Cfx (~300× minimal inhibitory concentration – MIC), SPA-tagged ParC subunits were affinity purified (Supplementary Figure 1A, verified by MS), followed by strand-specific sequencing of covalently attached DNA fragments and their mapping on a reference *E. coli* genome. The detected Topo IV cleavage sites (TCSs) had an enrichment patterns observed previously for DNA gyrase cleavage sites (GCSs) (Sutormin et al., 2019) and expected for type-IIA topoisomerases. Enrichment at TCSs has a bimodal shape with a sharp 4-bp gap in the middle. As explained elsewhere, the numbers of 3′-ends (N3Es) of enriched and sequenced DNA fragments comprise the gap ‘walls’ and precisely mark the cleavage site (Figure 1A). In total, 835 unique TCSs were identified in the reference genome. Among them, 356 TCSs were observed in at least two out of three biological replicates (Supplementary Figure 1B and Supplementary Table 1). Typically, around a primary strong TCS several weaker satellite TCSs were observed (Figure 1A, red and black arrows).

To increase the specificity of Cfx treatment, a mutation in the *gyrA* gene leading to the S83L single amino acid substitution conferring resistance of DNA gyrase to fluoroquinolones was introduced by recombineering. Phenotypically, the mutant strain behaves as the parental strain, with the average cell length and DNA content statistically indistinguishable from it (Supplementary Figures 2A–C, raw quantification data in Supplementary Table 3), and similar supercoiling level (Supplementary Figure 2D). In agreement with published data (Bagel et al., 1999), compared to parental DY330 *parC-SPA* the mutation led to specific 32-fold increase of MIC to Cfx (Supplementary Figure 2E). A Topo-Seq experiment revealed that in the presence of 10 μM Cfx the amount of Topo IV TCSs in the *gyrA-S83L* mutant was ~8-times higher than in the parental *wt* strain. A total of 6,768 unique TCSs were identified, of which 3,072 were recovered in at least two biological replicates (Supplementary Figure 1C and Supplementary Table 1). 301 (85%) out of 356 TCSs detected in the wild-type *gyrA* strain were also observed in the *gyrA-S83L* mutant, indicating that the gyrase mutation did not significantly alter the basal pattern of Topo IV activity. These shared TCSs had an increased average enrichment and, likely, comprise a core set of strong sites of Topo IV activity in the *E. coli* genome (Figure 1B). 3,127 TCSs were replicated at least in two independent experiments, either for *wt* or a derivative mutant strain, formed a dataset for further analyses (Supplementary Table 1).

Positions of identified TCSs were compared with positions of GCSs identified with Cfx earlier (Sutormin et al., 2019). Only 178 cleavage sites were shared between Topo IV and DNA gyrase (5.7% of TCSs and 3.8% of GCSs). Low fraction of coincide cleavage sites indicates that Cfx-induced cleavage bias was not dominating the individual cleavage preferences of the studied topoisomerases.

### The Topo IV cleavage motif lacks signs of DNA wrapping

The single-nucleotide resolution of Topo-Seq allowed us to identify the Topo IV cleavage motif. In addition to fluoroquinolone-specific nucleotide bias previously observed with Topo-Seq for DNA gyrase [more frequent cleavage before G nucleotides (Sutormin et al., 2019)], sequences flanking TCSs had several symmetrically enriched positions (−4G/+8C, −2A/+6 T, −1 T/+5A) previously reported as important cleavage determinants for Topo IV *in vitro* (Leo et al., 2005; Richter et al., 2007; Arnoldi et al., 2013; Figure 1C). Interestingly, these positions were also conserved for DNA gyrase (Supplementary Figure 3). The Topo-Seq data extended the Topo IV cleavage motif by revealing additional enriched positions −7C/+11G and −6C/+10G (Figure 1C). These positions were not conserved in the DNA gyrase motif (Supplementary Figure 3). It should be noted that the revealed Topo IV motif is rather weak and allows alternative nucleotides in most positions. On a broader scale, the Topo IV motif lacked any signs of periodic regions characteristic for the DNA gyrase motif (Sutormin et al., 2019) or any other motifs within 2 kb from the cleavage site, indicating that Topo IV is unlikely to wrap the immediate extensions of a G-segment DNA around its CTDs *in vivo* (Figure 1D).

### Topo IV activity in the Ter and Ori macrodomains

**Figure 2:**
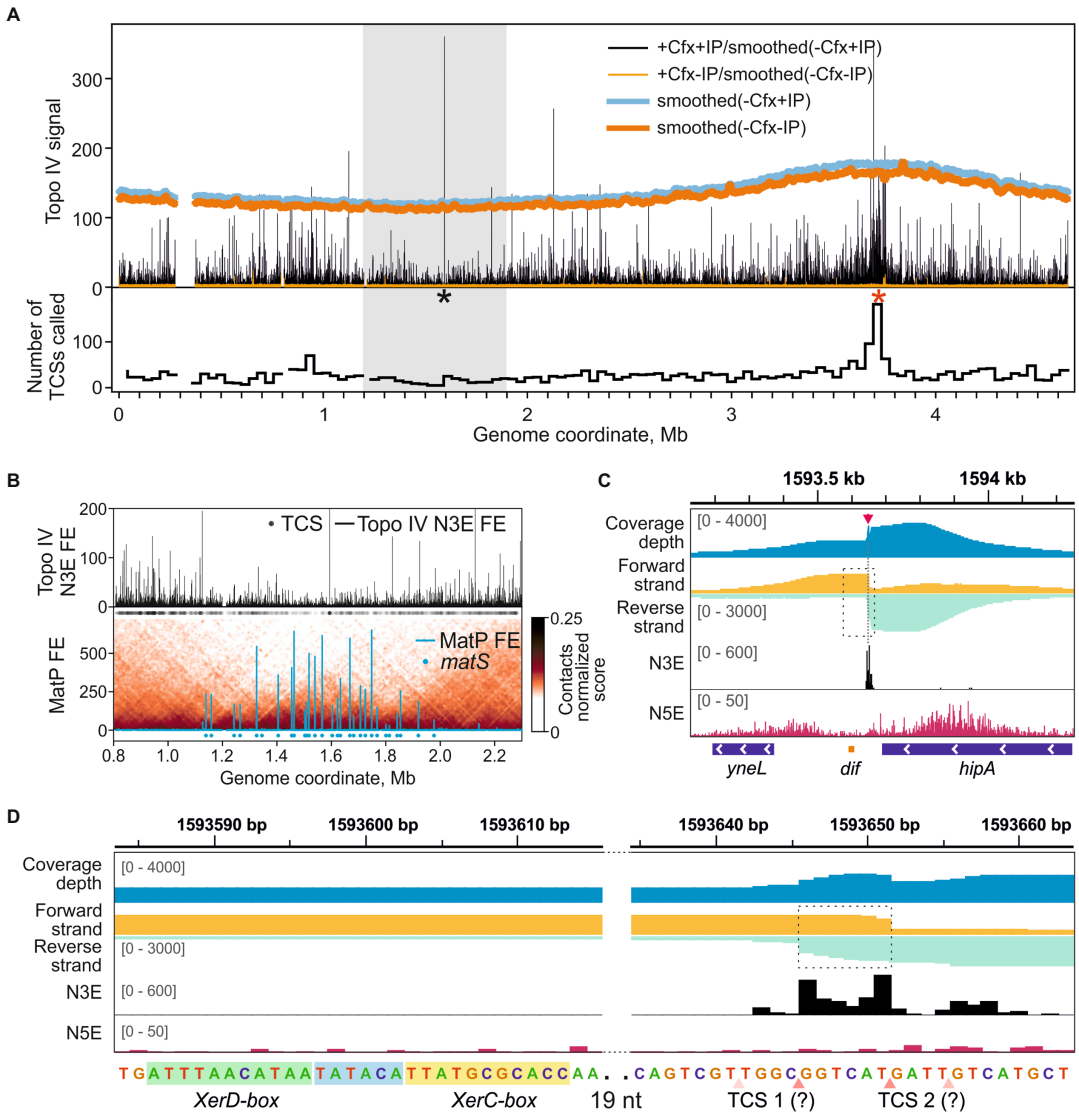
Topo IV cleavage activity at the *dif*-site. **(A)** Topo IV enrichment over the *E. coli* genome. Topo IV N3E fold enrichment (FE) for the +Cfx/+IP sample and the +Cfx/-IP control is shown in black and orange, respectively. Smoothed coverage depth tracks for -Cfx/+IP and -Cfx/-IP controls are shown in thick blue and dark orange curves, respectively. A black asterisk marks the position of the *dif*-site; red asterisk marks the position of *oriC*. A gray rectangle indicates the *E. coli* chromosome Ter MD. A plot representing the number of TCSs in 40 kb-wide genomic bins is shown below. **(B)** Topo IV N3E FE in the Ter macrodomain (upper plot). Black circles represent the positions of detected TCSs. MatP FE in Ter is shown below. Positions of *matS*-sites are shown with blue circles. Normalized contact map in Ter is shown in the underlying layer (resolution 10 kb). MatP data are taken from Nolivos et al. (2016), 3C contact data are taken from Lioy et al. (2018). **(C)** Topo IV enrichment and cleavage in the vicinity of the *dif*-site (orange rectangle below). N5E, N3E, and total coverage depth tracks are shown as in Figure 1A. Coverage depths for forward and reverse strands are shown in gold and turquoise, respectively. TCS is marked with a red arrow and a dashed line. A dashed rectangle indicates a region, containing the *dif-*site and the adjacent TCS, which is zoomed in **(D)**. The data are visualized in IGV (Thorvaldsdóttir et al., 2013). **(D)** Zoom-in of the *dif*-site region. Genomic sequence with marked XerD and XerC boxes is given below. Hypothetical asymmetric adjacent TCSs are shown with pink triangles below. Translucent triangles indicate the absent or depleted cleavage in the second DNA strand. Dashed rectangle marks the anomalously enriched region.

The sites of Topo IV cleavage activity revealed by Topo-Seq are non-uniformly distributed over the *E. coli* genome (Figure 2A). First, the Topo IV enrichment was depleted in the Ter MD (1.2–1.9 Mb marked with a gray rectangle in Figure 2A and zoomed-in in Figure 2B) with only 250 TCSs observed vs. 475 expected for this region (*p*-value 6.0e-34, binomial test). This depletion is in line with previous observations made with NorfIP (El Sayyed et al., 2016) and is likely mediated by the MatP protein which unloads the MukBEF-Topo IV complexes from the chromosome and is thought to coordinate sister chromosomes decatenation with cell division (Nolivos et al., 2016; Fisher et al., 2021). Indeed, the decrease in the Topo IV activity matched well with the region occupied by MatP (Figure 2B). This region also lacks high-range chromosomal contacts (Figure 2B; Nolivos et al., 2016; Lioy et al., 2018). Topo IV enrichment was gradually and symmetrically increased left and right of Ter MD, in so-called Left and Right MDs, respectively, and then decreased again (Valens et al., 2004). Specifically, average enrichments of TCSs in the 500-kb regions of Right and Left MDs adjacent to Ter MD were significantly higher than the enrichments of TCSs in the neighboring 500-kb regions (*p*-values 0.03 and 0.02, respectively; two-tailed *t*-test). This may indicate that active resolution of precatenanes occurs in the Left and Right MDs at the borders of the Ter MD. Gyrase cleavage enrichment was also depleted in Ter MD and increased in Left and Right MDs. However, unlike with Topo IV, gyrase enrichment did not show local maxima in these MDs and extended further toward the origin (Supplementary Figure 4; Sutormin et al., 2019).

One of the strongest Topo IV cleavage sites in the *E. coli* genome detected with Topo-Seq was located near the *dif*-site inside the Ter MD (Figures 2A,C). The site is recognized by the XerC/XerD recombinase. Previously, a TCS was mapped with low resolution *in vitro* in the vicinity of the *dif*-site (Hojgaard et al., 1999). Later, a strong cleavage at *dif* was detected *in vivo* using NorfIP (El Sayyed et al., 2016). However, the limited precision of the method did not allow to identify the exact position of the cleavage site. With Topo-Seq we detected two strong cleavage signals 33 and 38 bp away from the XerC-box (Figure 2D). The two N3E spikes were separated by 6 bp as observed in conventional cleavage sites (Figure 1A). Yet, sequenced fragments were not separated by a 4-bp gap but instead overlapped by 6 bp, which resulted in a local coverage peak in the center of a broader peak (Figure 2D). Such a pattern cannot be explained by a conventional double-stranded DNA cleavage by a single type-IIA topoisomerase complex or by extension of 3′-ends formed by Topo IV by DNA repair enzymes (the extension should have resulted in a 4-bp overlap with the two cleavage signals separated by 2 bp). We speculate that the observed pattern is formed by a superposition of two separate single-stranded cleavage events. Presumably, only reverse DNA strand is cleaved at one site, while the opposite, forward, DNA strand is cleaved at the other site, presumably by a different Topo IV complex. On a sequence level, the two cleavage sites did not resemble the derived Topo IV cleavage consensus, indicating that the enzyme was either attracted by the adjacent resolvase complex or that a different sequence recognition mode is realized at this site. There were no gyrase cleavage hot-spots either at *dif* or in the entire Ter MD in the presence of Cfx or other gyrase poisons (Supplementary Figure 4), indicating that the *dif* TCS is a highly specific target of Topo IV. However, visual analysis of gyrase enrichment profiles revealed a weak cleavage signal matching the position of Topo IV TCS at *dif* in either enriched or mock sequencing data for Cfx and oxalinic acid treated samples, but not for MccB17 treated samples (Supplementary Figure 5). The former two drugs, but not the latter one, inhibit Topo IV. This, and the fact that cleavage is observed in the absence of affinity purification suggests that the Topo IV induced cleavage at *dif* in the presence of fluoroquinolone poisons is so massive that it can be detected by sequencing genomic DNA without any enrichment.

Topo IV had an increased number of highly enriched cleavage sites near the *oriC* origin of replication (Figure 2A, a red asterisk). Overall, 950 TCSs were detected in the Ori MD vs. 622 TCSs expected (*p*-value 1.1e-16, binomial test). A particularly enriched region (251 TCSs vs. 42 expected, ~6-fold enrichment, *p*-value 1.1e-16, binomial test) spans 50–60 kb with *oriC* roughly in the middle. This enrichment was not observed by either ChIP-Seq or NorfIP earlier (El Sayyed et al., 2016). Interestingly, in the *wt* background the increased density of sites was less pronounced. Gyrase also had an increased number of GCSs both in the Ori MD and in the vicinity of *oriC*. This enrichment was less pronounced than that for Topo IV (Supplementary Figure 4) and is transcription-dependent (Sutormin et al., 2019).

### Topo IV is enriched downstream of active transcription units

**Figure 3:**
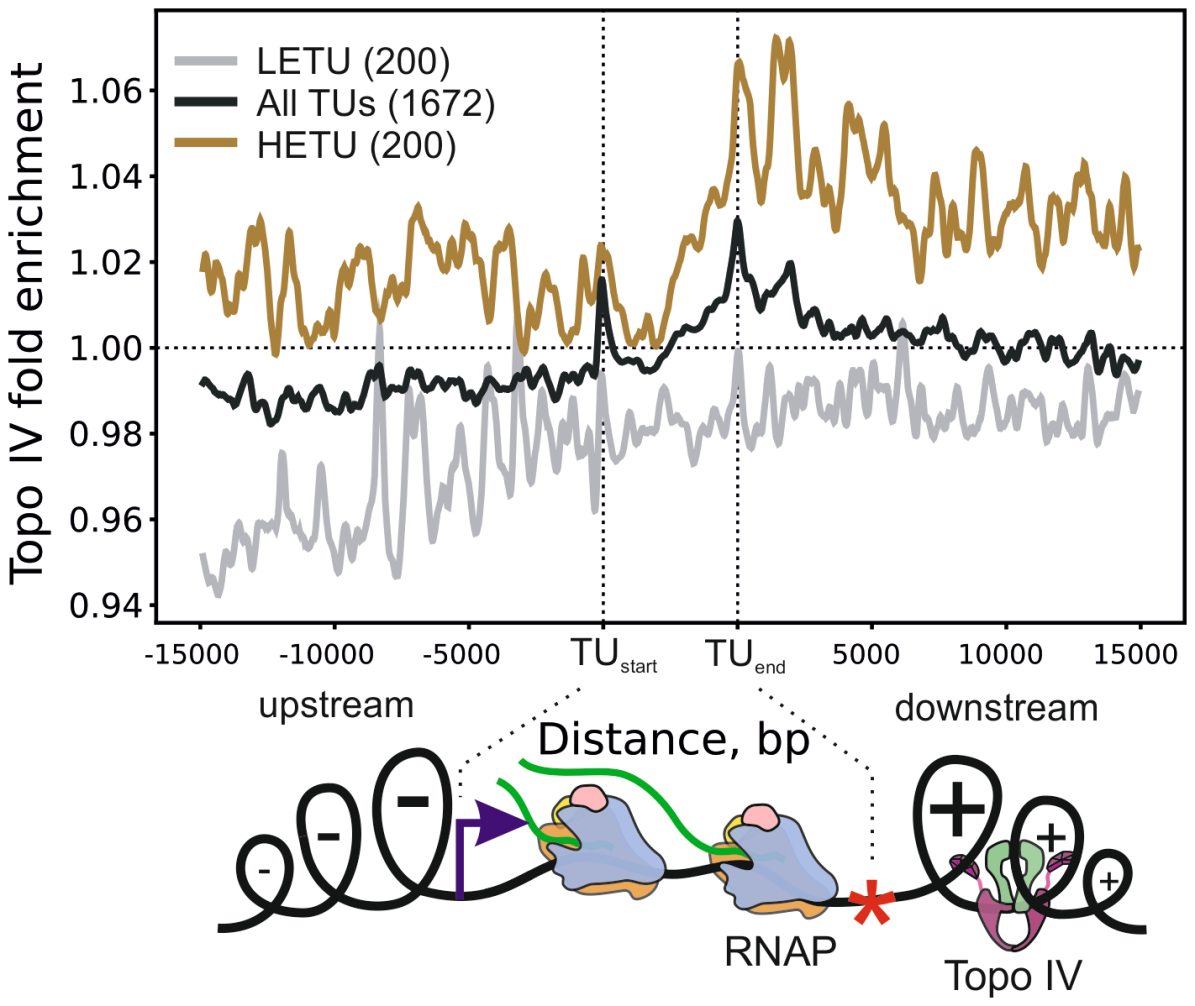
Metagene plot showing Topo IV enrichment in TUs, their upstream, and downstream regions. Enrichment is shown for all TUs (black curve), highly expressed (HETU, orange curve), and least-expressed (LETU, gray curve) sets. The number of TUs in each group is indicated in parentheses. Topo IV fold enrichment is given relative to the input sample (+Cfx + IP/-Cfx-IP). Bottom, graphical representation of the Liu and Wang twin-domain model (Liu and Wang, 1987) showing localization of RNAP and Topo IV. Data are shown for the *E. coli* DY330 *parC-SPA* strain.

To investigate the role of Topo IV in the relaxation of transcription-induced supercoiling, we analyzed the average enrichment of Topo IV in and around transcription units (TUs) stratified by their transcription level. Topo IV enrichment was decreased for least-expressed TUs (LETUs) and increased for highly expressed TUs (HETUs) (Supplementary Figures 6A,B). In addition, Topo IV enrichment was increased in HETUs downstream regions (Figure 3 and Supplementary Figure 6B), where positive supercoiling is accumulated (Sutormin et al., 2019; Guo et al., 2021; Visser et al., 2022). Especially high enrichment was observed in downstream regions of rRNA operons, which are highly transcribed in exponentially growing cells (Supplementary Figure 6C). Similar Topo IV enrichment was detected for both *wt* and gyrase mutant strains (Supplementary Figure 6D). Topo IV is known to interact with the MukBEF condensin and could have been expected to follow the MukBEF enrichment pattern if MukBEF enrichment was somehow affected by transcription or supercoiling (e.g., if elongating RNAPs move condensin to downstream regions). Indeed, on the level of enrichment sites, significantly more than expected TCSs were observed in MukB [a subunit of the MukBEF complex; MukB ChIP-Seq data from Nolivos et al. (2016)] enriched regions (938 TCSs vs. 648 TCSs expected, *p*-value 1.1e-16, binomial test). However, when we analyzed the MukB enrichment pattern relative to TUs, no comparable level of enrichment was observed downstream of HETUs (Supplementary Figures 6B,E). Therefore, the observed Topo IV enrichment pattern likely reflects the distinct properties of this topoisomerase and is unrelated to MukB. Overall, the Topo IV enrichment at TUs resembles enrichment pattern for DNA gyrase (Supplementary Figure 6F) indicating that Topo IV is also involved in relaxation of transcription-induced positive supercoiling. Correspondingly, correlations between Topo IV and DNA gyrase enrichments in 5 kb regions downstream of TUs were positive and significant (Pearson correlation coefficients 0.56 and 0.46 for *wt* and mutant strains, respectively), though Topo IV enrichment was less prominent and spanned over shorter distance compared to DNA gyrase enrichment (Supplementary Figures 6B,C). Interestingly, in contrast to DNA gyrase, Topo IV was not depleted in intergenic regions (compare Supplementary Figures 6D,F).

### Topo-Seq specificity and precision

**Figure 4:**
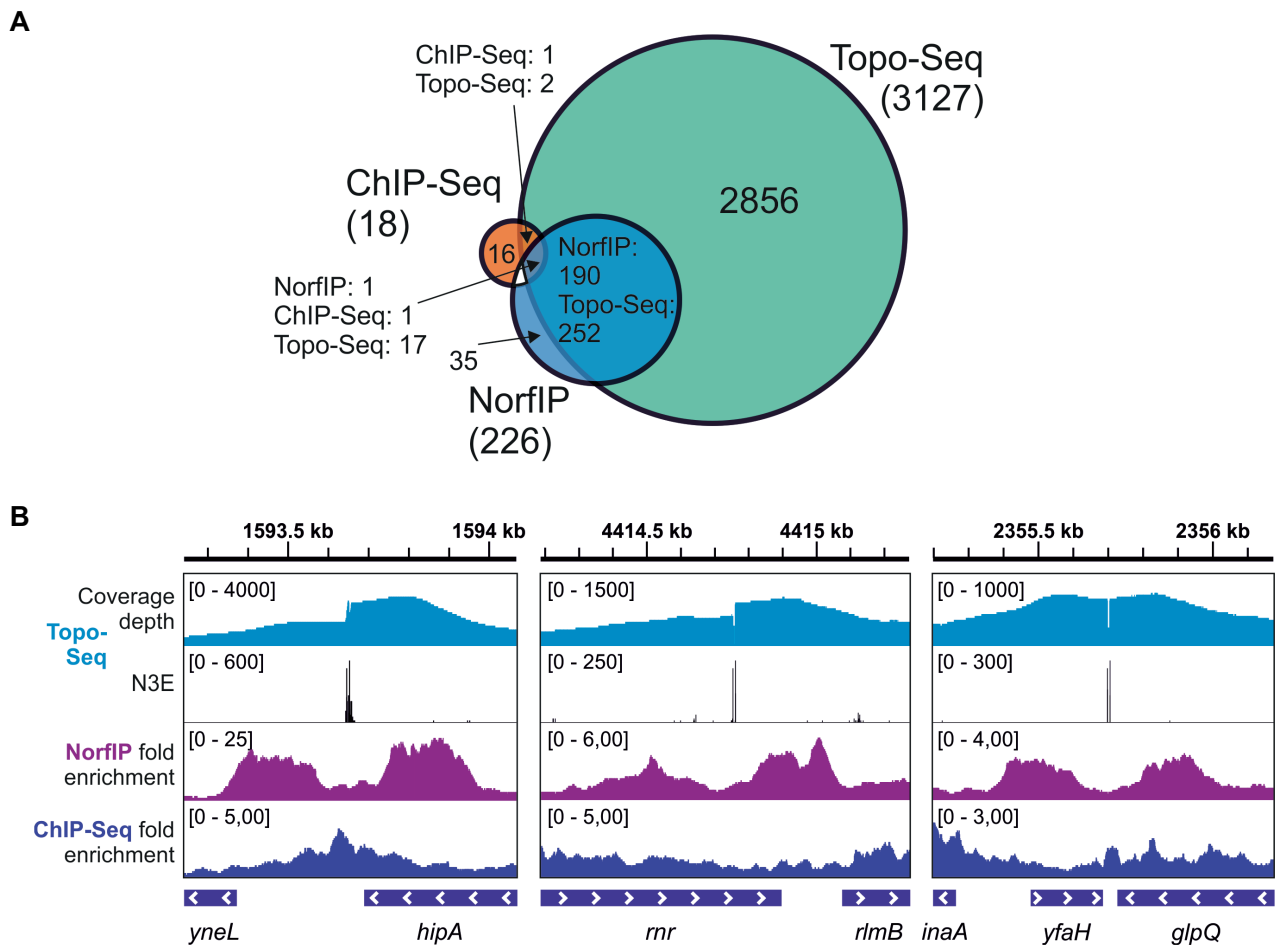
Sensitivity and precision of Topo-Seq. **(A)** A Venn diagram representing colocalization of Topo IV binding sites identified with ChIP-Seq and TCSs identified with NorfIP and Topo-Seq. Numbers of binding/cleavage sites are given for each subset, the total number of sites is shown in parentheses. **(B)** Representative genomic regions with binding/cleavage sites detected with ChIP-Seq, NorfIP, and Topo-Seq. ChIP-Seq and NorfIP data are taken from El Sayyed et al. (2016) and mapped to the *E. coli* reference genome used.

Previously, using NorfIP, a total of 571 Topo IV unique cleavage sites in the *E. coli* genome were identified in three independent experiments, with 234 common sites detected in at least two experiments, and 84 common sites detected in three experiments [(El Sayyed et al., 2016) and also see Supplementary Figure 7]. With Topo-Seq, the numbers of detected unique and doubly replicated TCSs were ~ 10 times higher (6,872 and 3,127, respectively), and the number of TCSs observed in at least four experiments (301) was ~3.5 times higher. The majority (84%) of Topo IV TCSs detected with NorfIP (190 out of 226 TCSs mapped to the reference genome used in this work) were colocalized with TCSs detected with Topo-Seq. The higher number of detected TCSs and a large fraction of NorfIP TCSs replicated with Topo-Seq confirms superior sensitivity of cleavage sites detection by our method (Figure 4A). In addition, while with NorfIP a typical binding/cleavage signal spanned several hundreds of bp, Topo-Seq identified TCSs with single-nucleotide resolution (Figure 4B). The increased sensitivity of Topo-Seq might be caused by differences in treatment (15 min with 10 μM Cfx for Topo-Seq vs. 10 min with 2 μM norfloxacin for NorfIP) which should lead to more efficient trapping of Topo IV at our conditions, and by a more efficient method for sequencing library preparation from modified or damaged DNA (Accel NGS 1S for Topo-Seq vs. standard Illumina TruSeq for NorfIP).

Only 19 regions were found to be significantly enriched with Topo IV by ChIP-Seq (El Sayyed et al., 2016), a method that does not distinguish between the binding and cleavage. Colocalization between the binding regions identified by ChIP-Seq and TCSs was limited (Figure 4). This result indicates that Topo IV cleavage occurs in only some binding regions (El Sayyed et al., 2016).

## Discussion

Using Topo-Seq, we identified several thousand Topo IV TCSs in the *E. coli* genome, which expands the repertoire of previously detected TCSs by almost an order of magnitude. We found that Topo IV enrichment pattern in the *E. coli* genome differs significantly from that of another type-II topoisomerase, the DNA gyrase. Topo IV cleavage is depleted in the Ter MD (except for strong and highly atypical cleavage at the *dif*-site), and gradually increases toward the Left and Right MDs. Additionally, Topo IV was strongly enriched in the Ori region.

We applied Topo-Seq to *wt E. coli* strain and for a derivative containing a *gyrA-S83L* mutation that confers resistance for fluoroquinolones. Surprisingly, sensitivity of Topo-Seq was much higher in the derivative strain, resulting in ~8-times more TCSs detected. Several independent observations suggest that the *gyrA-S83L* mutation by itself does not affect supercoiling and has a neutral effect on cell fitness (Aleixandre et al., 1989; Hallett and Maxwell, 1991; Marcusson et al., 2009). Likewise, our *gyrA-S83L* strain was phenotypically similar to *wt* and did not alter genome topology, DNA segregation, and plasmid supercoiling levels in our experimental conditions (Supplementary Figure 2). However, other reports suggest that substitutions of the GyrA S83 residue slightly reduce the supercoiling activity of the enzyme *in vitro* (a 10% decrease, Webber et al., 2017) and plasmid supercoiling *in vivo*, as judged by activity of a reporter gene controlled by supercoiling-sensitive promoter (a 14–22% decrease, Bagel et al., 1999). Such a decrease in gyrase activity, if it happened in our *gyrA-S83L* strain, could have been compensated by increased Topo IV activity. This effect, and increased availability of Cfx molecules unable to bind the mutant DNA gyrase might be jointly responsible for the appearance of additional Topo IV TCSs in the gyrase mutant background.

The single-nucleotide resolution of Topo-Seq and the large number of detected TCSs allowed us to identify the cleavage motif of *E. coli* Topo IV. As expected from observations made *in vitro* for fluoroquinolone-induced DNA cleavage by purified *E. coli* and *S. pneumoniae* Topo IV enzymes (Leo et al., 2005; Richter et al., 2007), Cfx induced strong preference toward cleavage before G nucleotides (Figure 1C). Consensus sequences of cleavage sites determined *in vitro* for *S. pneumoniae* Topo IV in the presence of Cfx and gemifloxacin (Gfx) (Leo et al., 2005; Richter et al., 2007) share significant similarity with the Topo-Seq-derived motif for the *E. coli* enzyme. Moreover, sites of Ca^2+^-induced cleavage (a method considered more native) had similar cleavage determinants (−4G/+8C, −2A/+6 T) suggesting intrinsic properties of the detected motif (Arnoldi et al., 2013). It should be noted that a consensus sequence obtained previously *in vitro* for *E. coli* Topo IV and the Topo-Seq-derived motif, seem to lack similarity between flanking sequences. The *in vitro* motif is shorter and lacks specificity determinants at positions −4/+8, −2/+6, probably, due to a lower sensitivity of the approach (Figure 1C). However, if one considers weaker nucleotide associations revealed *in vitro*, the two motifs become similar (Richter et al., 2007). With Topo-Seq, we further extended the Topo IV cleavage motif with −7C/+11G, −6C/+10G positions. The *E. coli* DNA gyrase cleavage motif determined *in vitro* and *in vivo*, by Topo-Seq, has similar cleavage determinants (−4G/+8C, −2A/+6 T, −1 T/+5A) indicating the conserved mode of G-segment recognition by type-IIA topoisomerases. −7C/+11G and −6C/+10G positions enriched in the Topo IV motif and absent in the gyrase motif, may thus determine Topo IV cleavage specificity. The overall motif of Topo IV is much shorter than DNA gyrase motif, lacks the periodic flanking regions, and generally resembles the eukaryotic Top2 motif (Gittens et al., 2019). The absence of periodic regions confirms that the ParC CTD does not interact with proximal extensions of G-segment DNA (Peng and Marians, 1995) but rather binds segments of crossing over DNA, which should favor resolution of precatenanes and catenanes. Differences between identified Topo IV and DNA gyrase motifs might contribute to the low fraction of cleavage sites that coincide for Topo IV and DNA gyrase.

With metagene analysis, we demonstrated that Topo IV is enriched in downstream regions of highly active TUs. This observation indicates that similarly to DNA gyrase, Topo IV is involved in relaxation of transcription-induced positive supercoiling *in vivo* which is in line with its *in vitro* preferences (Crisona et al., 2000; Ashley et al., 2017). Enrichment of Topo IV in the downstream regions of active TUs was lower than the DNA gyrase enrichment, indicating a limited contribution of Topo IV to the resolution of transcription-induced supercoiling, which is in accordance with the previous observations (Zechiedrich et al., 2000). Topo IV may act as a partial backup of DNA gyrase in this process. We found no clear colocalization between MukB and Topo IV in the downstream regions of active TUs. We speculate that Topo IV may act independently of MukBEF in these regions. Likely, Topo IV interacts with a plectonemic DNA formed by excessive positive supercoiling, as no G-segment DNA wrapping was indicated by Topo IV motif. Correspondingly, inhibition of transcription with rifampicin was shown to abolish norfloxacin-induced DNA cleavage by Topo IV (El Sayyed et al., 2016). To test this model directly, a Topo-Seq may be performed when the MukBEF-Topo IV interaction is uncoupled or when transcription is inhibited by rifampicin. In contrast to DNA gyrase, Topo IV was not depleted in intergenic regions. Probably, this reflects the difference between the motifs recognized by the two enzymes: DNA gyrase has a long and GC-rich motif which is depleted in the AT-rich intergenic regions, while Topo IV has a less constrained DNA motif.

We observed a strong depletion of Topo IV activity in the Ter MD, likely reflecting the depletion of MukBEF-Topo IV complexes by MatP (Nolivos et al., 2016; Burmann et al., 2021; Conin et al., 2022) and overall reduced transcription activity of this region. Why should MukBEF-Topo IV be displaced from Ter, and Topo IV activity be limited to the *dif*-site? It was proposed that an unloading system based on *matS*-MatP is required to recycle MukBEF associated with DNA and promote its uploading at the replication origin (Nolivos et al., 2016). We speculate that Topo IV depletion may prevent knotting and catenating of over-replicated chromosomes when the two replisomes fuse and travel further, replicating opposite replichores (Syeda et al., 2020). If so, an increased fraction of abnormal cells in *ΔmatP* mutants might have signs of DNA over-replication in the Ter region. In line with this model, linearization of *E. coli* chromosome in Ter by *tos*/TelN corrects both the *ΔmatP* phenotype and DNA over-replication in the Ter region (El Sayyed et al., 2016; Midgley-Smith et al., 2019). To decipher how transcription and MatP affect the activity of Topo IV in the Ter MD, more Topo-Seq data are needed, e.g., with mutant cells lacking MatP and cells treated with RNA polymerase inhibitor rifampicin. Interestingly, the depletion of gyrase enrichment in Ter MD was not affected by rifampicin (Sutormin et al., 2019), indicating that MatP may play a role in this phenomenon directly or indirectly, e.g., by unloading of MukBEF or constraining DNA supercoiling diffusing into the MD from the adjacent regions.

Topo IV has a well-known cleavage hot-spot near the *dif*-site (Hojgaard et al., 1999; El Sayyed et al., 2016), which we here map precisely. The cleavage site has a non-conventional structure and, likely, is a composite of two single-strand cleavage sites separated by 6 bp. Sterically, it is unlikely that two Topo IV complexes can simultaneously attack such two closely adjacent DNA sites. The cleavage events may happen independently on separate DNA molecules (e.g., on sister chromosomes) or occur probabilistically, giving a mixed signal in a bulk method like Topo-Seq. If so, then, only a single-stranded break will be introduced into DNA duplex by a hypothetical structurally or functionally asymmetric complex. Such a break is unable to support the conventional strand passage mechanism to decatenate sister chromosomes but may efficiently resolve supercoiling *via* the nicking-closing mechanism demonstrated *in vitro* for DNA gyrase heterocomplex with a single catalytic tyrosine (Gubaev et al., 2016). Relaxation of excessive DNA supercoiling [generated by elongating DNA polymerase (Postow et al., 2001) and/or translocating FtsK (Saleh et al., 2005)] might be important in Ter MD for proper replication termination and segregation of the replicated chromosomes. Consistently, in cells with a linearized chromosome, Topo IV does not cleave at *dif* despite the presence of all components of the *dif*-XerC/XerD ensemble, which implies that a topological stress is required for Topo IV activity at this site (El Sayyed et al., 2016). The proposed single-strand cleavage model conflicts with evidence of norfloxacin-induced double-stranded DNA cleavage detected at *dif* by Southern blot (Hojgaard et al., 1999; El Sayyed et al., 2016). Probably, the constitutive single-strand break mediates increased fragility of the region which results in increased breakage during DNA extraction and processing steps. Additionally, our model implies the existence of a mechanism that controls the Topo IV cleavage activity and stops it after the first DNA strand is cleaved. Alternatively, an observed cleavage pattern may be introduced by a non-canonical Topo IV complex consisting of two Topo IV molecules, which, cleaving together, introduce a double-strand break. Overall, Topo IV cleavage at *dif*-site represents an unusual behavior that is likely specifically orchestrated by its protein partners—XerC and FtsK—and may play a specific biological function that remains to be defined.

With Topo-Seq we observed an increased density of highly enriched TCSs at the Ori region. The locally increased cleavage activity may reflect the action of supramolecular MukBEF-Topo IV complexes recruited to Ori and required for proper segregation of this region once replicated (Nicolas et al., 2014; Nolivos et al., 2016). We speculate that Topo IV may be involved in early steps of replication progression where its activity can be critical to resolve precatenanes when two replisomes are relatively close to each other. In line with this speculation, MukB interaction with Topo IV was shown to stimulate relaxation of only negatively supercoiled DNA (Vos et al., 2013). Likely, the interaction with MukBEF *in vivo* provides specificity for DNA precatenanes formed by replication, since they form of a right-handed plectoneme—a topological equivalent of negatively supercoiled DNA in a plectonemic form (Schvartzman et al., 2019). Increased transcriptional activity in the Ori region might also cause the accumulation of Topo IV activity, similarly to what has been observed for DNA gyrase (Sutormin et al., 2019). Topo-Seq experiments with cells treated with rifampicin may help to investigate the role of transcription in this phenomenon.

ChIP methods provide essential information on the genomic localization of DNA-binding proteins. The classical method is based on formaldehyde fixation of protein-DNA complexes, however, it suffers from low sensitivity, high background noise, and low precision (Waldminghaus and Skarstad, 2010; Chen et al., 2012). A plethora of methods such as Native ChIP, ChIP-Exo, CUT&RUN, CUT&TAG, etc., were developed to overcome these limitations (Ma et al., 2019; Leo and Romano, 2021). For topoisomerases, which naturally form covalent adducts with DNA during the catalytic cycle, using specific poisons or mutations, intermediate complexes can be stabilized and enriched without the crosslinking agent (McKie et al., 2020). Topoisomerase adducts, however, should be completely removed before sequencing, otherwise it may interfere with library preparation steps and reduce the precision of the method (El Sayyed et al., 2016). As we demonstrated here for *E. coli* Topo IV and previously for DNA gyrase and TopoI (Sutormin et al., 2019, 2022), Topo-Seq has a high sensitivity and a single-nucleotide resolution which makes it a method of choice for detection of topoisomerase cleavage sites in various model systems.

## Data availability statement

The datasets presented in this study can be found in online repositories. The names of the repository/repositories and accession number(s) can be found in Supplementary Table 2.

## Author contributions

DS conceived the study, analyzed data, and prepared figures. DS, AGal, and AGaf performed the experiments. DS and KS wrote the draft and finalized the manuscript. All authors contributed to the article and approved the submitted version.

## Funding

This work (experiments and bioinformatic analysis) was supported by a grant from the Ministry of Science and Higher Education of the Russian Federation (agreement No. 075-10-2021-114 from 11 October 2021). This work was also supported by Skoltech NGP Program (Skoltech-MIT joint project) and RFBR grant, project number 20-34-90069. Sequencing at Skoltech Genomics Core Facility was supported by the Skoltech Life Sciences Program grant.

## Conflict of interest

The authors declare that the research was conducted in the absence of any commercial or financial relationships that could be construed as a potential conflict of interest.

## Publisher’s note

All claims expressed in this article are solely those of the authors and do not necessarily represent those of their affiliated organizations, or those of the publisher, the editors and the reviewers. Any product that may be evaluated in this article, or claim that may be made by its manufacturer, is not guaranteed or endorsed by the publisher.

## Footnotes

^1^ https://github.com/sutormin94/Topo IV_Topo-Seq_experiment

^2^ https://github.com/axelcournac/EColi_analysis

^3^ https://github.com/axelcournac/3C_tutorial
