# Supplementary material for: Single-nucleotide resolution detection of Topo IV cleavage activity in the Escherichia coli genome with Topo-Seq
Source: Front Microbiol. 2023 Apr 6;14:1160736. doi: 10.3389/fmicb.2023.1160736 (PMC10117906; doi:10.3389/fmicb.2023.1160736)
Supplement: Supplementary file 4 [file Data_Sheet_1.doc]

***Supplementary materials***

**Single-nucleotide resolution detection of Topo IV cleavage activity in the *E. coli* genome with Topo-Seq**

**Dmitry Sutormin^1^, Alina Galivondzhyan^1^, Azamat Gafurov^1^, Konstantin Severinov^3*^**

^1^Skolkovo Institute of Science and Technology, 121205 Moscow, Russia

^3^Waksman University for Microbiology, Rutgers, NJ 08854, USA

*** Correspondence:**Konstantin Severinov
[severik@waksman.rutgers.edu](mailto:severik@waksman.rutgers.edu)

Correspondence may also be addressed to Dmitry Sutormin. E-mail: [D.A.Sutormin@gmail.com](mailto:D.A.Sutormin@gmail.com)


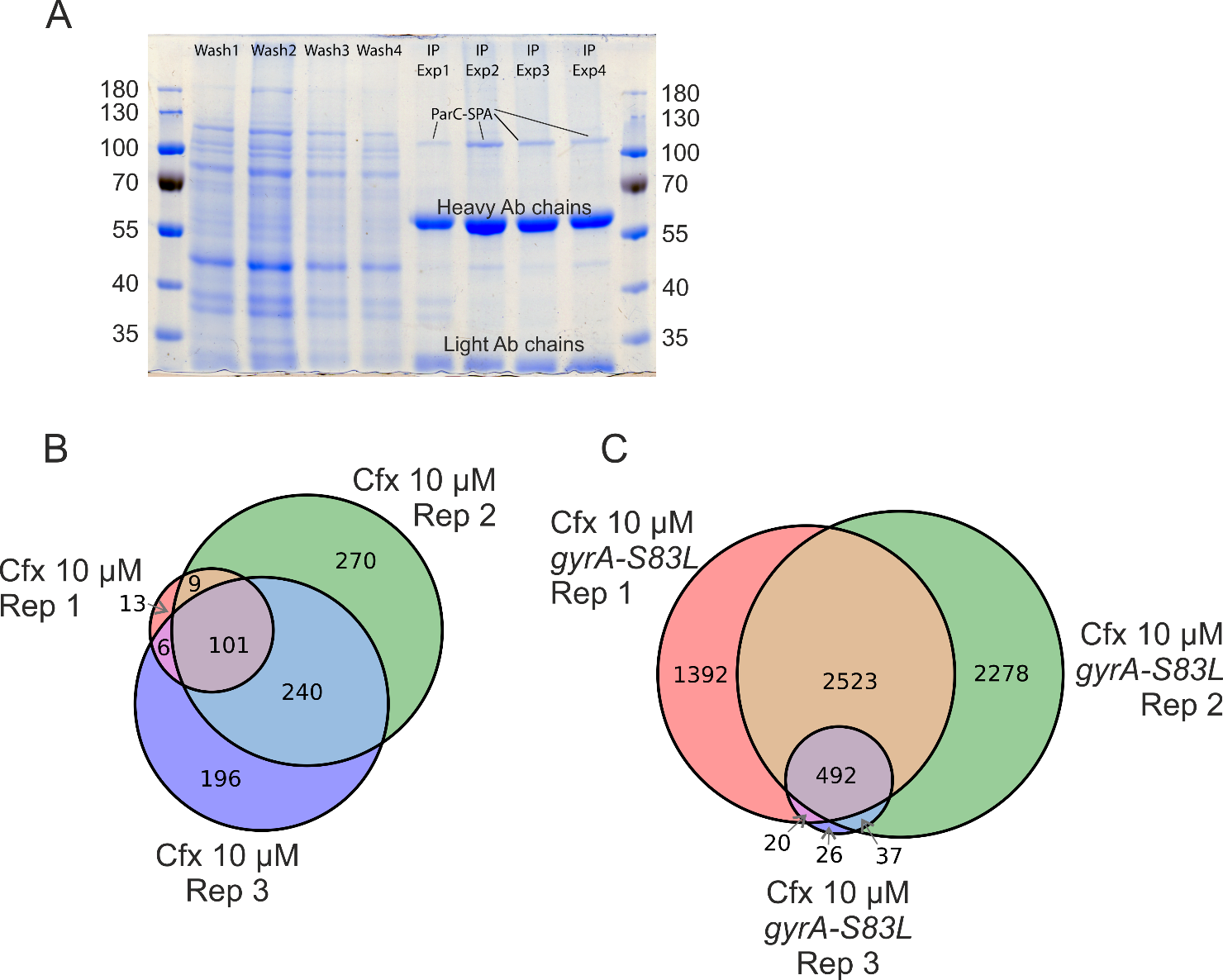


**Supplementary Figure 1. (A)** Coomassie-stained PAGE of immunoprecipitation stages of Topo-Seq for Topo IV. Four consecutive wash fractions are shown and the resultant IP fractions with enriched ParC-SPA subunits. Experiments 1 and 2 were performed with *E. coli* DY330 *parC-SPA* and Experiments 3 and 4 were performed with *E. coli* DY330 *parC-SPA* *gyrA-S83L*. Marker weights shown in kilodaltons. **(B)** A Venn diagram representing the overlaps between sets of topoisomerase cleavage sites determined in Topo-Seq replicates with the *E. coli* DY330 *parC-SPA* strain. **(B)** A Venn diagram representing the overlaps between sets of topoisomerase cleavage sites determined in Topo-Seq replicates with the *E. coli* DY330 *parC-SPA* *gyrA-S83L* strain.

**
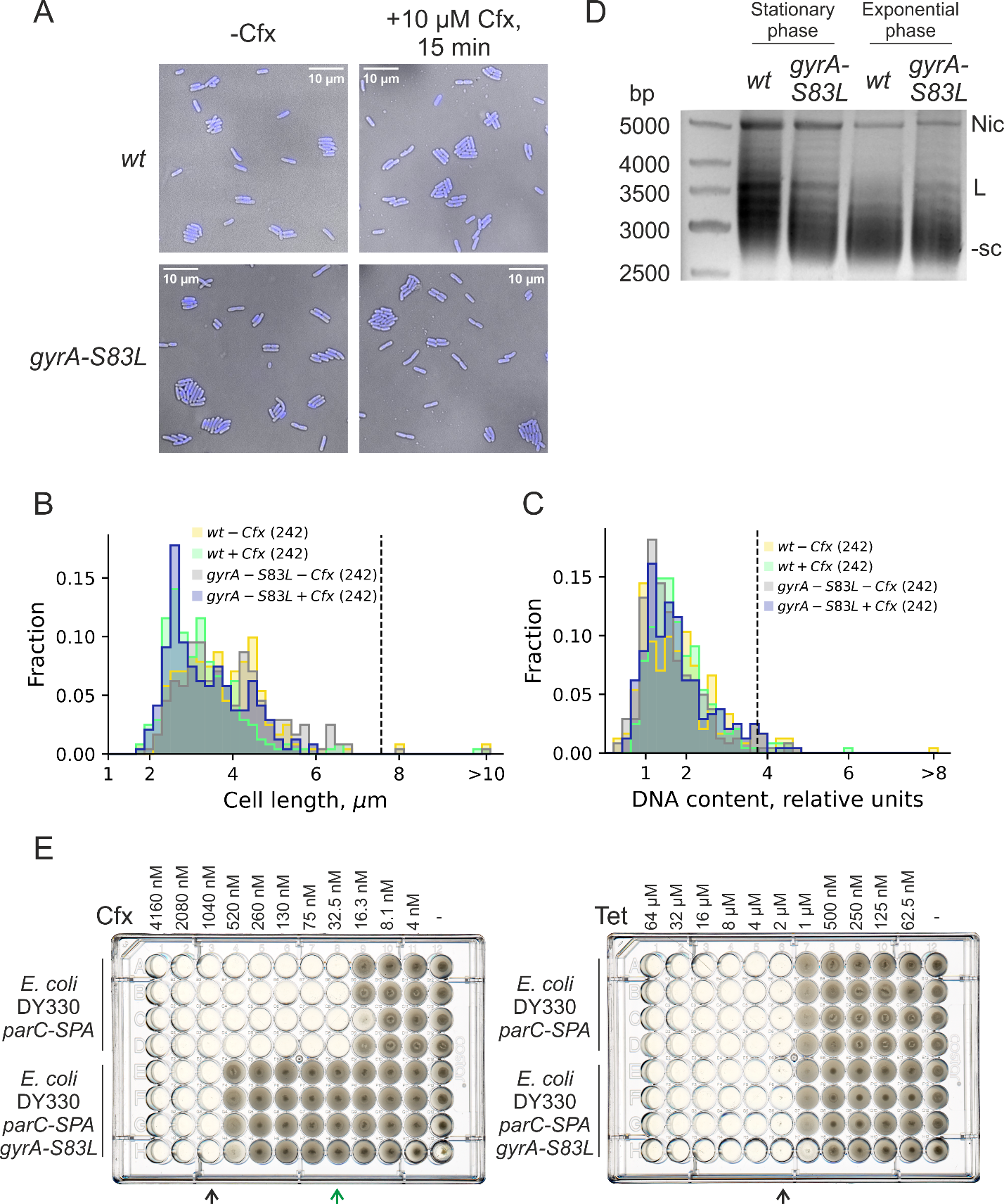
**

**Supplementary Figure 2. (A)** Microphotographs of *E. coli* DY330 *parC-SPA* and *E. coli* *DY330 parC-SPA gyrA-S83L* cultures treated and not-treated with Cfx. Cells were stained with DAPI. **(B)** Quantification of cell length in cultures described in panel (A). Cells >10 µm are collected into an overflowing bin. The number of cells is indicated in parentheses. Source data can be found in Supplementary Table 3. **(C)** Quantification of cells DNA content in cultures described in panel (A). Cells with a DNA content >8 relative units are collected into an overflowing bin. The number of cells is indicated in parentheses. Source data can be found in Supplementary Table 3. **(D)** Supercoiling of pMP1000 plasmid extracted from stationary-phase and exponentially growing E. coli DY330 parC-*SPA* and E. coli DY330 parC-*SPA gyrA-S83L* strains. Nic - nicked plasmid, L - linear plasmid, −sc - negatively supercoiled plasmid. 2.5 µg/mL chloroquine was added to separate topoisomers. **(E)** Titration plates for MIC determination for ciprofloxacin (left) and tetracycline (right). MICs were measured for *E. coli* DY330 *parC-SPA* and *E. coli* DY330 *parC-SPA gyrA-S83L* strains in four biological replicates. Arrows below indicate the MICs.

**
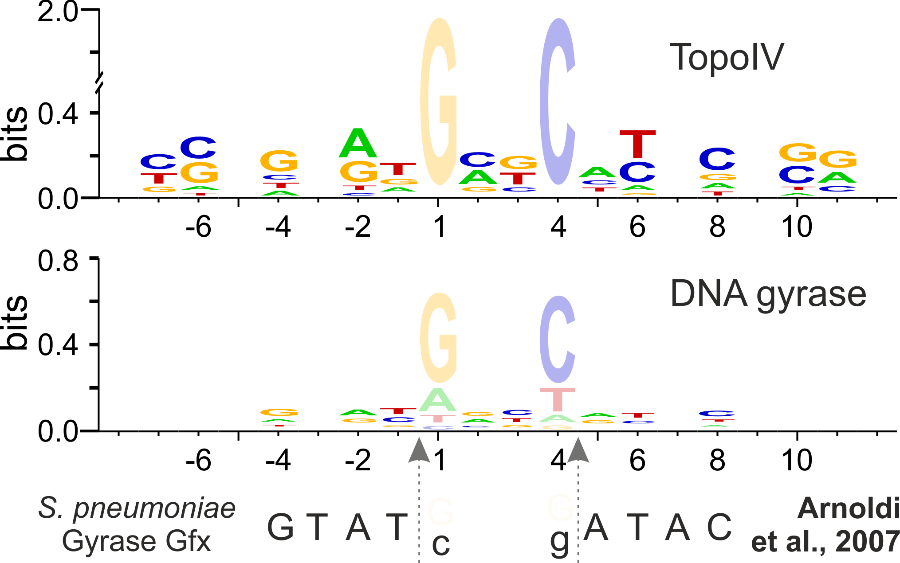
**

**Supplementary Figure 3.** Logo representation of Topo IV and DNA gyrase motifs determined with Topo-Seq. Date for DNA gyrase is taken from (Sutormin et al., 2019). Cfx-biased positions at 0 and +3 are shown translucent. For Topo IV logo G and C at 0 and +3 are shown not to scale. Below the logos, cleavage consensus sequences identified previously for *S. pneumonia* DNA gyrase *in vitro* are shown (Arnoldi et al., 2013). Preferred bases are shown in capital letters and non-preferred bases are shown in lowercase letters. Gfx – gemifloxacin. Vertical dashed arrows indicate cleavage sites positions.


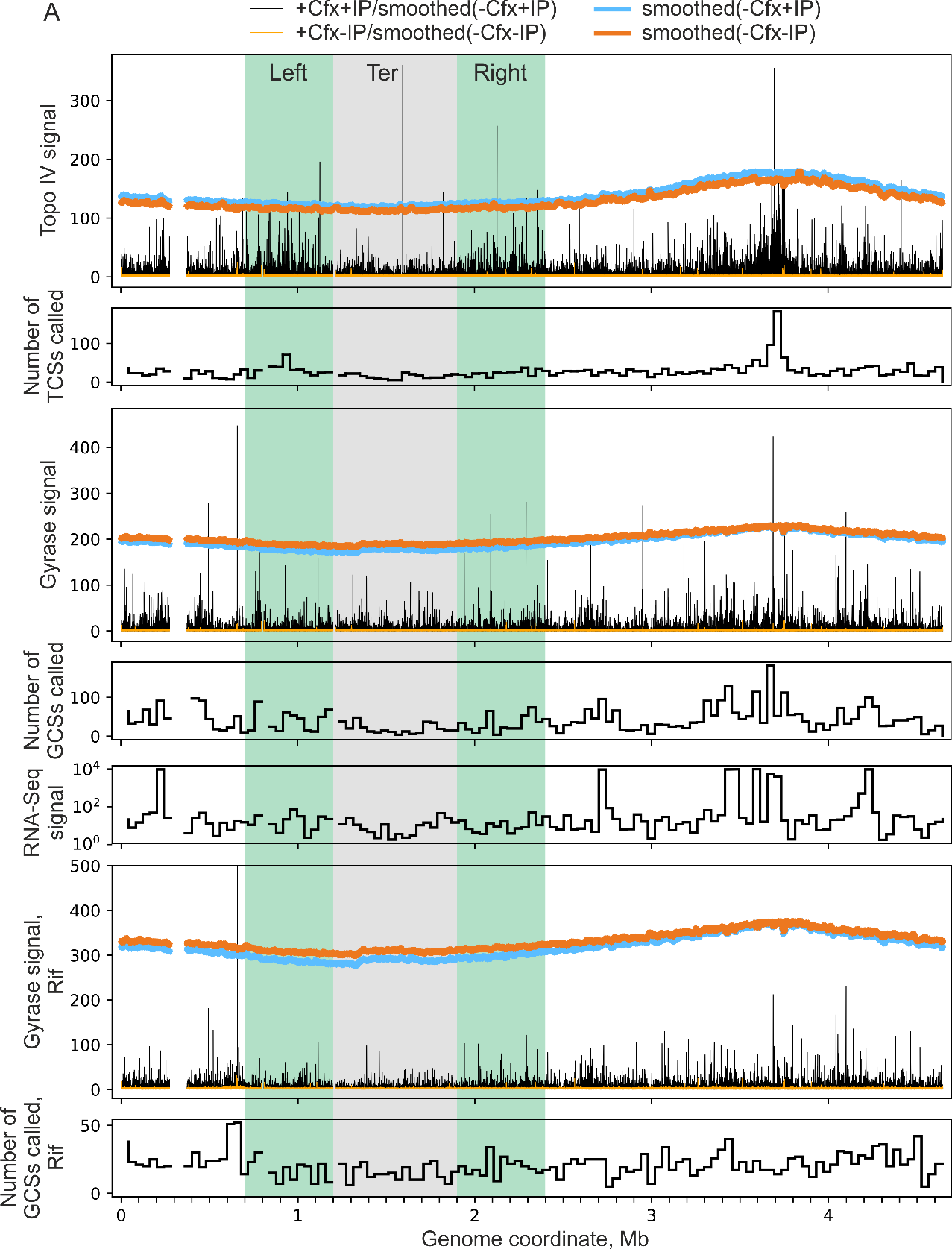
**Supplementary Figure 4.** Comparison of Cfx-induced Topo IV cleavage activity with DNA gyrase cleavage activity (Cfx), DNA gyrase cleavage activity in cells pre-treated with rifampicine (RifCfx), and RNA-Seq data. Cleavage activity and numbers of cleavage sites are shown as in Figure 2A. RNA-Seq data was binned correspondingly. Gyrase cleavage data was taken from (Sutormin et al., 2019), RNA-Seq data was taken from (Sutormin et al., 2022). Ter MD is shown as a grey zone, two regions left and right of Ter with gradually increasing Topo IV signal are shown as green zones.


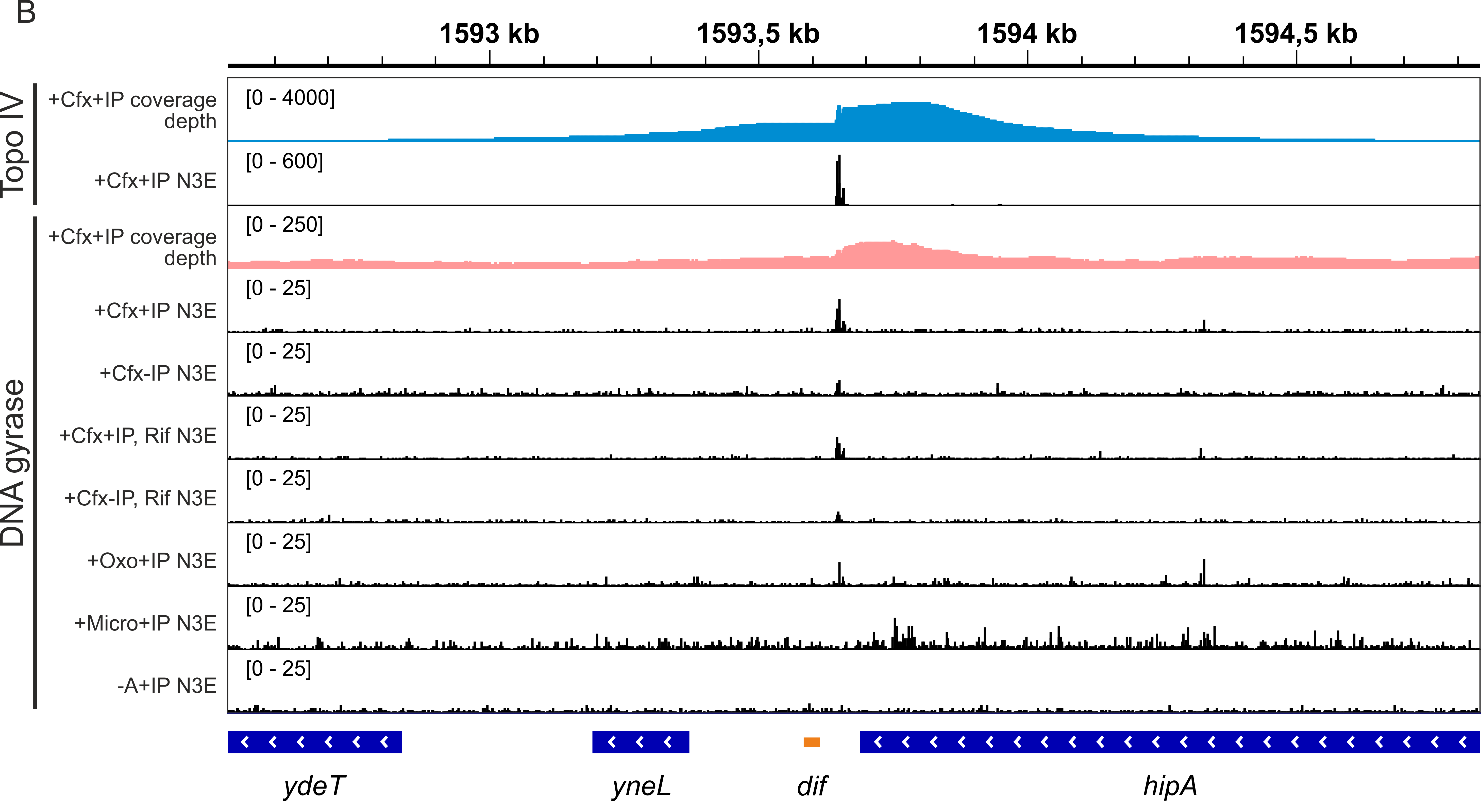


**Supplementary Figure 5.** Comparison of Cfx-induced Topo IV cleavage activity with DNA gyrase cleavage activity in a *dif*-site region in the presence of different poisons: Cfx, RifCfx, Oxo (oxolinic acid), and Micro (microcin B17). Background cleavage in the absence of any poison is shown (-A). N3E tracks are shown for enriched (+IP) and mock (-IP) samples.


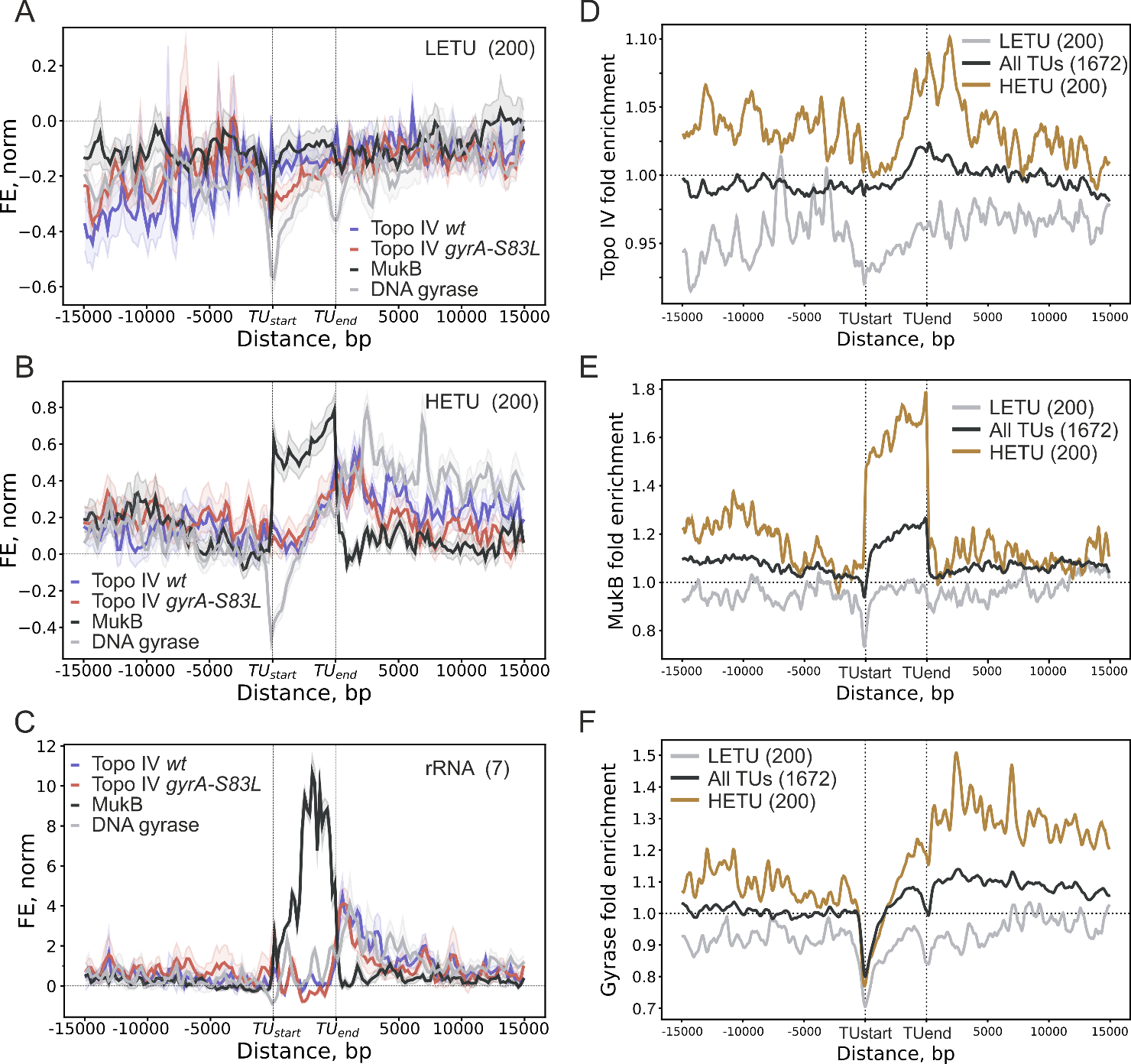


**Supplementary Figure 6.** Metagene plot showing normalized Topo IV (from the *E. coli* DY330 *parC-SPA* – referred as *wt* – and the *E. coli* DY330 *parC-SPA gyrA-S83L* – referred as *gyrA-S83L* – strains), MukB, and DNA gyrase enrichments in TUs, their upstream and downstream regions for LETUs **(A)**, HETUs **(B)**, and rRNA operons **(C)**. Confidence bands (±SEM) in panels (**A)-(C)** are represented by light-colored profiles. Metagene plot showing not-normalized Topo IV (from the *E. coli* DY330 *parC-SPA gyrA-S83L* strain) **(D)**, MukB **(E)**, and DNA gyrase **(F)** enrichments in TUs, their upstream and downstream regions. Enrichment is shown for all TUs (black curve), highly-expressed (HETU, orange curve), and least-expressed (LETU, grey curve) sets. The number of TUs in each group is indicated in parentheses. Topo IV and DNA gyrase fold enrichments are given relative to the corresponding input samples (+Cfx+IP/-Cfx-IP). Topo IV enrichment data for Topo-Seq performed on a *E. coli* *wt* strain. MukB ChIP-Seq data was taken from (Nolivos et al., 2016), DNA gyrase Topo-Seq data was taken from (Sutormin et al., 2019).


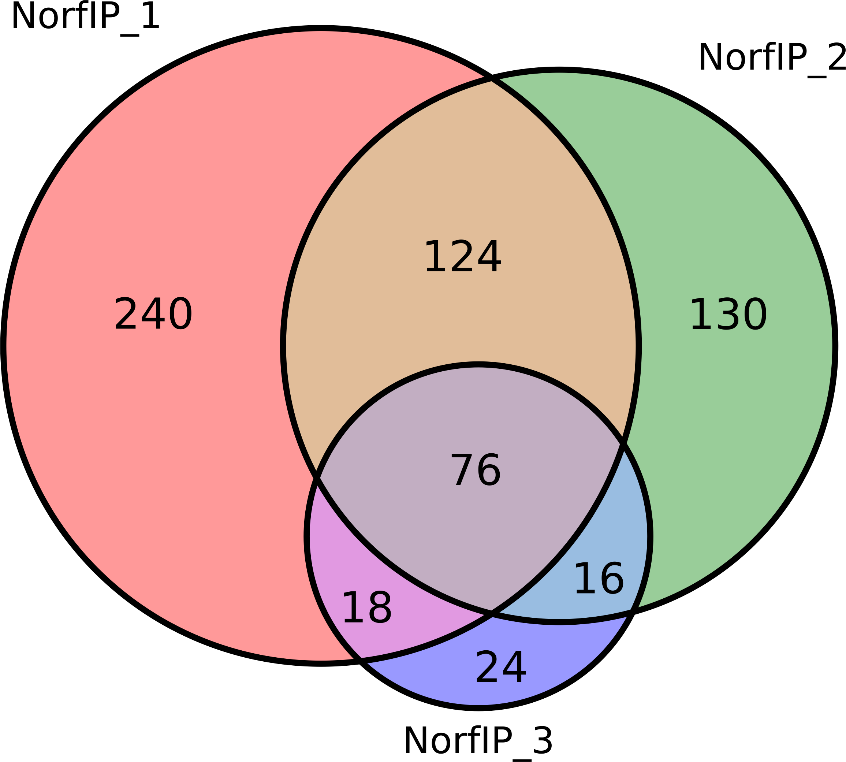


**Supplementary Figure 7.** Venn diagram representing the overlaps between sets of topoisomerase cleavage sites determined in NorfIP replicates. TCSs identified at least in two replicates out of three, were considered for further analysis. Data was taken from (El Sayyed et al., 2016).

**References**

Arnoldi, E., Pan, X., and Fisher, M. (2013). Functional determinants of gate-DNA selection and cleavage by bacterial type II topoisomerases. *Nucleic Acids Res.* 41, 9411–9423. doi: 10.1093/nar/gkt696.

El Sayyed, H., Le Chat, L., Lebailly, E., Vickridge, E., Pages, C., Cornet, F., et al. (2016). Mapping Topoisomerase IV Binding and Activity Sites on the E. coli Genome. *PLoS Genet.* 12, 1–22. doi: 10.1371/journal.pgen.1006025.

Nolivos, S., Upton, A. L., Badrinarayanan, A., Müller, J., Zawadzka, K., Wiktor, J., et al. (2016). MatP regulates the coordinated action of topoisomerase IV and MukBEF in chromosome segregation. *Nat. Commun.* 7. doi: 10.1038/ncomms10466.

Sutormin, D., Galivondzhyan, A., Musharova, O., Travin, D., Rusanova, A., Obraztsova, K., et al. (2022). Interaction between transcribing RNA polymerase and topoisomerase I prevents R-loop formation in E . coli. *Nat. Commun.* 13, 1–19. doi: 10.1038/s41467-022-32106-5.

Sutormin, D., Rubanova, N., Logacheva, M., Ghilarov, D., and Severinov, K. (2019). Single-nucleotide-resolution mapping of DNA gyrase cleavage sites across the Escherichia coli genome. *Nucleic Acids Res.* 47, 1–16. doi: 10.1093/nar/gky1222.
